# Engineering protein nanocages as carriers for biomedical applications

**DOI:** 10.1038/am.2016.128

**Published:** 2017-04-07

**Authors:** Sathyamoorthy Bhaskar, Sierin Lim

**Affiliations:** School of Chemical and Biomedical Engineering, Nanyang Technological University, Singapore,

**Keywords:** Biomaterials - proteins, Biomaterials - proteins

## Abstract

Protein nanocages have been explored as potential carriers in biomedicine. Formed by the self-assembly of protein subunits, the caged structure has three surfaces that can be engineered: the interior, the exterior and the intersubunit. Therapeutic and diagnostic molecules have been loaded in the interior of nanocages, while their external surfaces have been engineered to enhance their biocompatibility and targeting abilities. Modifications of the intersubunit interactions have been shown to modulate the self-assembly profile with implications for tuning the molecular release. We review natural and synthetic protein nanocages that have been modified using chemical and genetic engineering techniques to impart non-natural functions that are responsive to the complex cellular microenvironment of malignant cells while delivering molecular cargos with improved efficiencies and minimal toxicity.

## Protein nanocages: Versatility beyond natural functions

Researchers have identified three components of self-assembled protein nanocages (PNCs)that can be engineered to improve delivery of molecular cargos. Biological systems such as virus capsids and the protein ferritin have cage-like structures and membrane-permeating capabilities that make them natural platform candidates for transporting molecules directly to cells. Sierin Lim and her team from Nanyang Technological University in Singapore survey progress in modifying PNCs interiors to improve drug loading, exteriors to enhance biocompatibility and display of ligands, and interactions between subunits to tune molecular release. Hybrid nanostructures that combine protein scaffolds with synthetic components, such as thermoresponsive polymers, are helping to impart additional functions to the PNCs. The authors foresee PNCs that combine multiple functionalities on a single platform and their development as agents in immunotherapy and immunoengineering as future directions for the field.

## Abstract (web summary)

Formed by the self-assembly of protein subunits, protein nanocages can be engineered at the interior, exterior, and inter-subunit locations. Each type of modification can be tuned for specific biological applications. Multiple-modifications can be imparted to these nanocages without compromising its self-assembling property, creating multi-functional protein nanocage platforms. Potential applications of the modified protein nanocages include targeted delivery in complex cellular environments for therapeutic and diagnostic agents as well as modulation of cellular responses.

## Graphical abstract

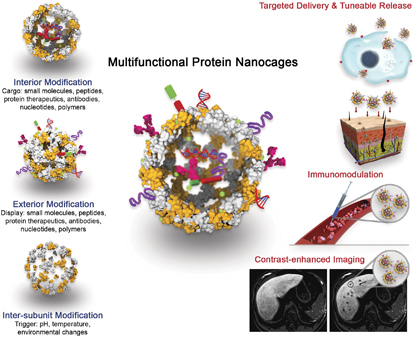

## Introduction

Smart nanosized materials with high stability, suitable pharmacokinetics and efficient cell permeability for delivering cargo molecules to target cells have been the requirements for an ideal drug delivery system.^1^ Advances in the design and fabrication of synthetic carriers such as cationic liposomes, micelles, block copolymers, carbon nanotubes, dendrimers and inorganic nanoparticles are restricted by severe toxicity and low delivery efficiency.^2^ Nature-derived nanocarriers are potential alternatives to synthetic ones as they satisfy most of the key features, such as biocompatibility, water solubility and high cellular uptake efficiency with minimal toxicity.^1, 3^ Examples of nature-derived nanocarriers include protein nanocages such as viruses, ferritin and many others that are formed by the self-assembly of protein subunits, resulting in a cage-like structure. The monodispersed subunits are modifiable through chemical and genetic methods.^3^ The key biomedical applications involving the use of protein nanocages that we focus on in this review are therapeutics and diagnostics.

Among myriad nature-derived nanocarriers, protein-based biological systems such as viruses have been a subject of intense study owing to their innate ability to penetrate cell membranes. The structure of viruses best represents the principles of protein assembly in nature. Structural analyses of viruses show that they consist of a protein shell comprising a definite number of subunits that surrounds and protects its genome.^4^ Viral capsids are naturally programmed for host-cell targeting and cell entry. They have evolved to mediate the exchange of nucleic acids between different chemical environments.^5^ Viruses are stable structures that have the ability to withstand environmental pressures but are sensitive enough to detect signals or the change in signals in cellular environment, thereby releasing the nucleic acids they carry in the target microenvironment. Viruses have thus been an inspiration in developing diverse self-assembling protein nanocages from natural sources. Important aspects of protein nanocages such as biocompatibility, functional diversity, biological fabrication and flexibility of design by protein engineering make them powerful materials for various applications.^5^ Viruses have been engineered to perform specific functions. For example, bacteriophages have been used in peptide display, filamentous phages have been used as templates for nanofabrication and virus-like particles (VLPs) have been used as immunogens. With the exception of peptide-displaying filamentous phages commonly used for nucleic acid and conjugated drug delivery *in vitro* and *in vivo*, these nanoparticles are hardly suitable for cell-targeted drug delivery.^6^

Specific functional polypeptides can be modified to self-assemble into nanoparticles with or without caged structures with desirable nanoscale properties in terms of size and geometry. The assembly of the functional building blocks to form protein nanoparticles, as observed in natural viruses, can seldom be mimicked by general nanofabrication techniques.^6^ The intricacy stems from selecting protein sequences that promote protein–protein interactions without nonspecific aggregation. These protein sequences can be derived from both natural and non-natural amino-acid sequences that lead to self-assembly in various patterns.^6^ Natural protein scaffolds are structures that already exist in nature with intrinsic self-assembling properties, including viruses, ferritin and eukaryotic vaults. In contrast, synthetic protein scaffolds are designed *de novo* to mimic the properties of the natural scaffolds and carry out specific functions. The advantage of both natural and synthetic protein scaffolds is that they can be exploited for the design of novel functional architectures.^5^ Specific applications of protein nanocages for drug delivery have been discussed in a review by Molino *et al.*^7^ and Schoonen *et al.*^8^ In this review, we focus on natural and synthetic protein scaffolds engineered with specific functional groups to impart non-native functions, including aiding the delivery of active molecules through targeting of malignant cells and overcoming cellular barriers.

## Protein scaffolds

### Natural scaffolds

Protein scaffolds have been found to facilitate the formation of various inorganic structures in nature. For example, the single-celled algae *Emiliania huxleyi* form fine calcium carbonate structures called coccoliths. Proteins control the formation of these structures by guiding the assembly of crystallites into three-dimensional structures.^4^ An examination at the molecular level shows that complex systems such as cells use proteins, in addition to phospholipids, as physical boundaries that define and separate the inside of the cell into subcellular compartments. Here, we present five classes of structures that are distinct in size and morphology: viruses, ferritin, E2 protein, vaults and other protein nanocages.

#### Viruses and virus-based particles

**Figure 1:**
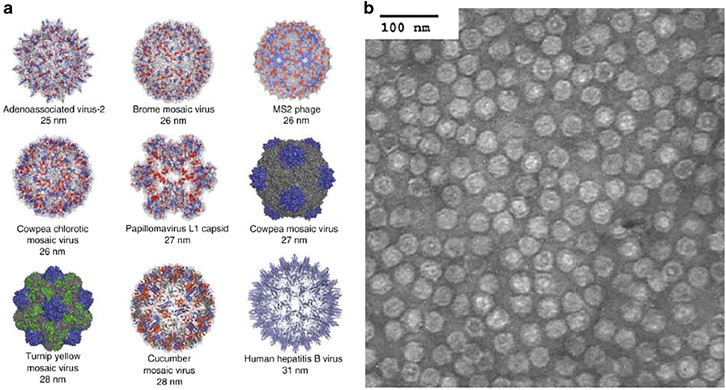
(**a**) Structural illustration of viruses used in bionanotechnology and biomedical applications.^3^ (**b**) Purified turnip yellow mosaic virus (TYMV) particles negatively stained with uranyl acetate, scale bar=100 nm^3^ (reprinted from Lee, L.A. & Wang, Q. Adaptations of nanoscale viruses and other protein cages for medical applications/virology. *Nanomedicine* 2, 137–149. Copyright (2006), with permission from Elsevier).

Years of studies to deduce information about viral assembly, replication and infection pathways provide a clear idea on the stability and functionality of viruses.^3^ Viral transduction has been observed to be facilitated by self-assembling protein structures called capsids, which interact with host cells and deliver their genome into the target cell compartment.^1^ The outer surface of the viral capsid opens up several opportunities for diverse chemical functionalization and site-specific modifications to synthesize organic/inorganic materials and to link targeting moieties.^9^ Viruses have an innate mechanism for protecting their genome, transferring it to the extracellular environment, escaping the immune system upon reaching target cells by interacting with their receptors, and delivering the packaged nucleic acids into the desired cell compartment. Therefore, viruses are used as functional carriers for targeted delivery with built-in advantages over synthetic delivery vehicles.^1^ Viruses are the dominant class of the bionanoparticle family, with sizes ranging widely from 10 to 100 nm (Figure 1).^3^ In addition to cell targeting ability and gene delivery efficiency, these protein-based multifaceted systems have highly ordered spatial configurations, and the stability and functionality of these materials have already been established through intensive research with advances in understanding virus infection, replication and assembly pathways.^3^

The cowpea mosaic virus (CPMV) (PDB ID 2BFU) was the first nanocage among viruses shown to be conjugated to a variety of functional molecules. This nanocage is a well-characterized plant virus that can be produced in high amounts. The composition of the amino-acid residues in the virus’s structure is well understood for bioconjugation with functional molecules. The virus is stable over a wide range of pH levels, temperatures and organic solvents. It could therefore be used for assembling other inorganic nanoparticles.^10^ Genetically engineered CPMV possessing thiol groups are produced and covalently attached to gold nanospheres. The nanospheres self-assemble into icosahedral plasmonic nanoclusters following the locations of the thiol groups on the CPMV and exhibit a 10-fold increase in local electromagnetic fields. These plasmonic nanoclusters have unique optical properties that can be used for spectroscopy and cancer treatment.^10^ Multiple orthogonal reactive sites on viral nanocages can be established by genetic engineering for bioconjugation with signalling moieties and biological recognition motifs.^11^ Two different amino-acid residues of turnip yellow mosaic virus (PDB ID 1AUY) are used for conjugation to luminescent terbium complexes and biotin molecules. The modified nanocages can be used as a scaffold for the development of sensor by time-resolved fluoroimmunoassay.^11^ Recent advances in selective chemical modifications of viruses and viral-based particles have broadened the scope of modulating bionanoparticles beyond genetic mutations.^3^

#### Ferritin

In 1937, ferritin was discovered as a novel protein structure for storing and transporting iron molecules. Ferritin isolated from horse spleen contained 20% iron in its native structure.^9^ Subsequently, it has been found to exist almost ubiquitously in biological systems, regulating the storage and release of iron to maintain homeostasis. These findings have shed light on the idea that proteins can be used to encapsulate and deliver active molecules *in vivo*. The first X-ray diffraction data of horse spleen ferritin were reported in 1943^12^ and the structure in 1984,^13^ but it was not until 1991 that the crystal structure of human ferritin (1FHA) was first reported and the cage-like architecture was clearly elucidated.^14^ Since then, numerous ferritins and ferritin-like proteins have been developed for exogenous applications other than iron storage.^9^

**Figure 2:**
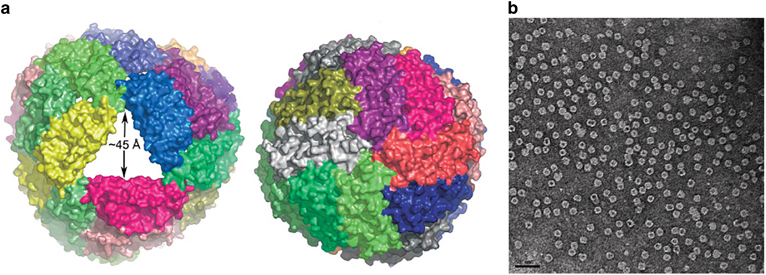
(**a**) PyMOL illustrations of the tetraeicosameric assembly observed in *Archaeoglobus fulgidus* ferritin viewed along different molecular symmetry axes. Left: One of four pores of the nanocage structure shown along the threefold noncrystallographic symmetry axis. Right: Engineered closed ferritin shell viewed along the axis the fourfold molecular symmetry axis^15^ (reprinted from Johnson, E., Cascio, D., Sawaya, M.R., Gingery, M. & Schroder, I. Crystal structures of a tetrahedral open pore ferritin from the hyperthermophilic archaeon *Archaeoglobus fulgidus*. *Structure* 13/4, 637–648. Copyright (2005), with permission from Elsevier). (**b**) Transmission electron micrograph of engineered *A. fulgidus* ferritin, scale bar=50 nm^16^ (reprinted from Sana, B., Johnson, E., Sheah, K., Poh, C.L. & Lim, S. Iron-based ferritin nanocore as a contrast agent. *Biointerphases* 5/3, FA48–FA52. Copyright (2010), with permission from Springer).

The globular, multisubunit caged ferritin stores iron in an insoluble non-toxic state while keeping it bioavailable within cells by readily converting it to its soluble form. The iron in the central cavity is maintained as small crystalline particles in an organic ferric oxyhydroxide form. Ferritin can withstand high temperature and a wide range of pH levels for limited periods without significant disruption to its caged quaternary structure, which is formed by 24 subunits. These subunits self-assemble into a hollow spherical structure with an outer diameter of 12 nm and an internal cavity with a diameter of 8 nm^5^ (Figure 2). Ferritin’s iron core is approximately the size of its internal diameter. This critical diameter and several molecular characteristics, including electrophoretic mobility, solubility and antigenicity, are the most significant properties of the protein shell.^13^ The self-assembly and disassembly properties of the ferritin nanocage can be controlled by metal ions, thereby aiding the uptake and release of active molecules from the protein structure.^17^ Similarly, the structural and biochemical properties of ferritin protein are used for tailoring it to a wide range of applications, from the synthesis of nanoparticles to the design of vaccines in biomedicine.^18^

#### E2 protein

**Figure 3:**
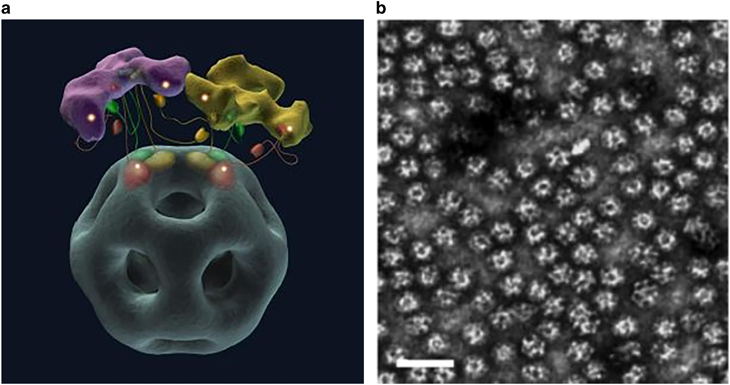
(**a**) E1-E2–E3 complex of *Geobacillus stearothermophilus* active site coupling model. Tetramers of E1 (purple) and dimers of E3 (yellow) are shown attached to the outer surface of icosahedral E2 protein (gray) formed by 60 subunits^19^ (reprinted from Milne, J.L. *et al.* Molecular structure of a 9 MDa icosahedral pyruvate dehydrogenase subcomplex containing the E2 and E3 enzymes using cryoelectron microscopy. *J. Biol. Chem.* 281, 4364–4370. Copyright (2006). with permission from The American Society for Biochemistry and Molecular Biology). (**b**) Electron micrograph of wild-type E2 protein, scale bar=50 nm^20^ (reprinted from Dalmau, M., Lim, S., Chen, H.C., Ruiz, C. & Wang, S.W. Thermostability and molecular encapsulation within an engineered caged protein scaffold. *Biotechnology and Bioengineering* 101, 654–664. Copyright (2008), with permission from John Wiley and Sons).

The E2 protein is derived from the E2 core domain (dihydrolipoamide acetyltransferase) of the pyruvate dehydrogenase multienzyme complex. The protein is formed by 24 subunits in *E. coli* or 60 subunits in *Geobacillus stearothermophilus* that self-assemble into a hollow structure with a cubic core or an icosahedron with twelve 5-nm openings (PDB ID 1B5S), respectively.^19^ Pyruvate dehydrogenase enzyme complex is composed of three enzymes: pyruvate decarboxylase (E1), dihydrolipoamide acetyltransferase (E2) and dihydrolipoamide dehydrogenase (E3) (Figure 3). It has been shown that the complex can be produced separately and the component enzymes can be reassembled *in vitro* to form the native structure.^19^ Because E1 and E3 do not dissociate from each other in the presence of the enzyme E2, it is evident that the latter forms the structural core of the assembled complex. *G. stearothermophilus* is a thermophilic organism. Hence, the pyruvate dehydrogenase complex derived from it has innate stability that allows it to survive under extreme conditions. Unlike other nanocages such as viral capsids, the modification of the external surface with functional ligands does not hinder the assembly of the E2 core domain. This property is identical to that in its natural state, in which the E2 domain is linked to two distinct folding protein domains at its surface.^19^ Similar to other natural protein nanocages, E2 protein nanocages can be subjected to genetic or chemical modifications to enable several functions such as drug loading and specific targeting protein attachment. Ren *et al.* showed that the E2 protein can be modified both on the internal and external surfaces for loading of drug molecules inside and for displaying functional epitopes on the outer surface of the nanocage simultaneously.^21^ Thus, the E2 protein cage offers promising avenues for tailored engineering of the exterior, the subunit–subunit interfaces and the interior to produce the desired modifications.^22^

#### Vaults

First reported in 1986, vaults are ribonucleoparticles with a mass of 13 MDa found in most eukaryotic cells.^23^ Vaults have a barrel-like structure measuring 41 × 41 × 72.5 nm^3^ with extending caps at their extremities and a hinged waist region.^24^ The nanostructure consists of several highly conserved proteins, including the major vault protein (MVP: PDB IDs 2QZV, 4V60), which constitutes over 70% of the overall mass of the nanoparticle, vault poly(ADP-ribose) polymerase (VPARP), telomerase-associated protein 1 (TEP1) and some untranslated RNA molecules.^25^ The basic vault structure consists of 78–96 MVPs arranged from the C to N terminus from the cap to the waist, forming a thin protein coat covering an internal cavity of 5 × 10^7^ Å^3^. The N terminus is tucked towards the interior of the vault. The MVPs are non-covalently associated. Despite the strong binding between MVPs, the vault has a dynamic structure that opens and closes transiently, a phenomenon referred to as breathing, to incorporate small molecules, proteins and other macromolecules within the inner core.^24^

**Figure 4:**
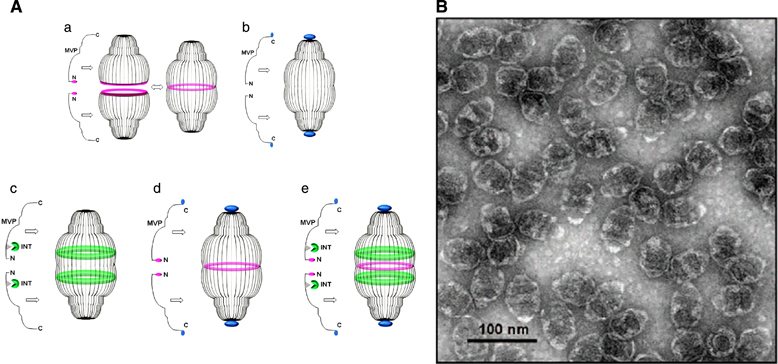
(**A**) Schematic representation of vault nanocages engineered by inclusion of additional amino acids at the N and C termini of major vault protein (MVP). Peptide extensions at the N termini (shown in red) and C termini (shown in blue) from the waist region (light red) and the extreme ends of the caps, respectively (**A**a and b). By fusing an exogenous protein at INT binding site (**A**c) and additional peptide at the N terminus (**A**d), a composite assembly with multifunctional units could be formed (**A**e)^26^ (reprinted (adapted) Han, M., Kickhoefer, V.A., Nemerow, G.R. & Rome, L.H. Targeted vault nanoparticles engineered with an endosomolytic peptide deliver biomolecules to the cytoplasm. *ACS Nano* 5, 6128–6137. Copyright (2011), with permission from American Chemical Society). (**B**) Transmission electron microscopy (TEM) image of recombinant vault particles, scale bar=100 nm^27^ (reprinted (adapted) from Kickhoefer, V.A. *et al.* Targeting vault nanoparticles to specific cell surface receptors. *ACS Nano* 3, 27–36. Copyright (2009), with permission from American Chemical Society).

A shuttle peptide, called INT, derived from the interaction domain located at the C terminus of the VPARP (GenBank accession no. AF158255; amino acid 1563–1724), attaches itself to the inner side of the vault shell through a unique protein–protein interaction (Figure 4). When fused to the interaction domain INT, non-native proteins will be shuttled to the interior of the vault, and the loaded proteins have been shown to retain their original characteristics with optimal stability under both biological and physical stress conditions.^27^ Recombinant production of MVPs using a baculovirus expression system in insect cells shows that the protein structure can sufficiently aid in directing the formation of recombinant vault particle with a structure analogous to the wild-type particles. The naturally occurring vault nanocages can thus be engineered for a wide range of novel applications.^28^

#### Other natural scaffolds

**Figure 5:**
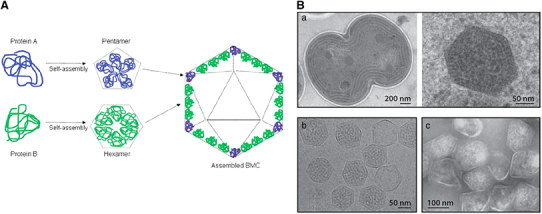
(**A**) Structural illustration of the formation of icosahedral bacterial microcompartments (BMCs). The BMCs are formed by hexameric (green) and pentameric (blue) folding proteins. The hexamers assemble side by side to form a flat molecular layer of the icosahedron; the vertices of the same structure are formed by pentameric proteins^1^ (reprinted from Ferrer-Miralles, N. *et al.* Engineering protein self-assembling in protein-based nanomedicines for drug delivery and gene therapy. *Crit. Rev. Biotechnol.* 1–13, 1549–7801. Copyright (2013), with permission from Taylor & Francis Ltd). (**B**a) TEM images showing the division of a cyanobacterial cell (left) and the structure of a carboxysomes (right). (**B**b) TEM image showing purified carboxysomes derived from *Halothiobacillus neopolitanus*. (**B**c) Purified Pdu microcompartments derived from *Salmonella enterica*^30^ (reprinted from Yeates, T.O., Crowley, C.S. & Tanaka, S. Bacterial microcompartment organelles: protein shell structure and evolution. *Annu. Rev. Biophys.* 39, 185–205. Copyright (2010), with permission from Annual Reviews).

Bacterial species have been found to host self-assembling nano- and microsized structures called bacterial microcompartments (BMCs) containing protein complexes formed by 60–20,000 copies of one or more self-assembled protein species. BMCs are part of specific metabolic pathways of the cell, encapsulating unstable or toxic intermediate metabolites.^1^ Carboxysomes were the first bacterial organelles identified to perform such functions. As described in 1973, carboxysomes have icosahedral structures with a cross-section measuring 100–150 nm and a protein coat containing 6–10 distinct proteins; they are involved in autotrophic CO_2_ fixation in bacteria via RuBisCO and carbonic anhydrase enzymes.^1^ Similar to viral capsids, the formation of carboxysomes’ icosahedral structure requires a combination of hexameric and pentameric self-assembling units of BMC proteins. Interestingly, the self-assembly property of the carboxysomes was retained *in vivo* even after the deletion of the RuBisCO enzyme.^29^ The flat sheet of the icosahedron is formed by the hexameric units that assemble side by side, whereas the vertices are occupied by the pentameric units (Figure 5). A common BMC subunit consists of 90 amino acids with an α-/β-fold structure.^1^ In 1994, a protein-based organelle from the hyperthermophilic bacterium *Thermotoga maritima*, referred to as ‘linocins’, was described as the smallest BMC, measuring 20–24 nm in diameter.^31^ Later, these protein structures were renamed ‘encapsulins’.^1^

### Synthetic bioinspired protein structures

Constant efforts are being made to engineer natural protein sequences to impart new or additional functions. Recent studies have taken the efforts to a higher level by creating self-assembling protein structures through *de novo* design. The assembly of small peptides occurs through various non-covalent interactions, van der Waals, hydrophobic, π–π staking, electrostatic interactions, hydrogen bonds and disulfide bond formation.^1^ On the smallest possible scale, small proteins that self-assemble to form helical bundles consisting of two to four helices are designed by focusing on simple folding patterns, particularly the α-helices or coiled-coil arrangements.^32^ The phenomenon of biological self-assembly observed in amyloidogenesis, in which a soluble protein is transformed into insoluble aggregates (amyloid fibrils) by a chemically specific molecular assembly process involving domain swapping and symmetric oligomerization, could be considered the inspiration in designing artificial supramolecular protein structures.^32^ Stringing multiple protein domains to construct modular proteins allows for the incorporation of multiple functions into a single polypeptide chain. The modular protein has been used for *in vitro* condensation and targeted cell delivery of nucleic acids.^1^ This novel approach has paved the way to designing a variety of protein/DNA complexes to deliver nucleic acid therapeutics both *in vitro* and *in vivo*.

**Figure 6:**
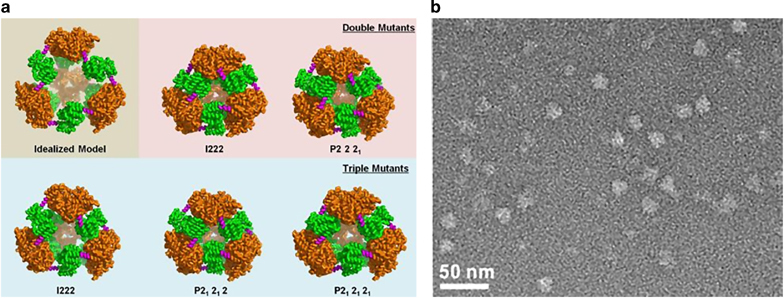
(**a**) Crystal structures of double and triple mutant-type protein nanocages formed by the oligomer fusion strategy. The trimeric domains are shown in orange, and dimeric domains are shown in green. (**b**) TEM image of the triple mutant form of the designed protein nanocage, scale bar=50 nm^35^ (reprinted (adapted) Lai, Y.T. *et al.* Structure and flexibility of nanoscale protein cages designed by symmetric self-assembly. *J. Am. Chem. Soc.* 135, 7738–7743. Copyright (2013), with permission from American Chemical Society).

**Figure 7:**
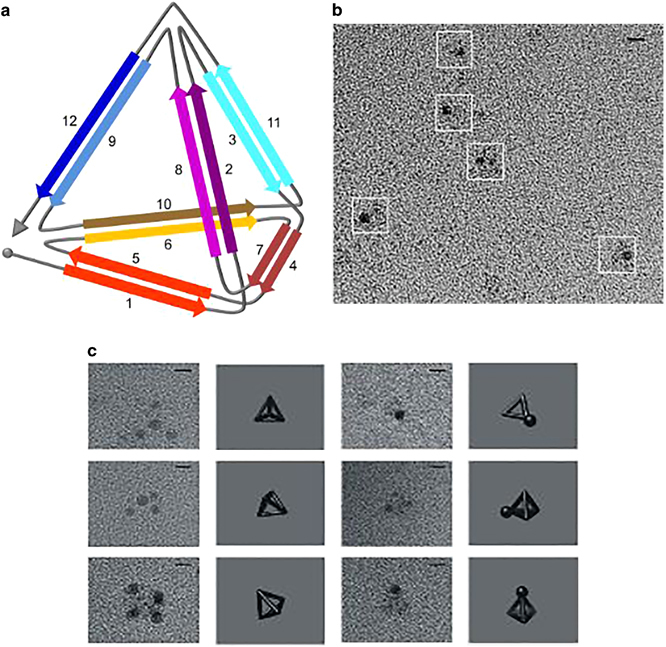
(**a**) Illustration of tetrahedron-shaped polypeptide structure. (**b**) Coil-forming elements in the polypeptide self-assembly are denoted by arrow marks. Transmission electron microscopy (TEM) image of TET12 tetrahedral polypeptide highlighted in white boxes. TEM images of TET12 structure (left) stained with 1.8 nm Ni-NTA nanogold beads followed by uranyl acetate staining, scale bar=5 nm. (**c**) On the right, TEM images of TET12 polypeptide from the white boxes shown in (**b**)^37^ (reprinted from Gradisar, H. *et al.* Design of a single-chain polypeptide tetrahedron assembled from coiled-coil segments. *Nat. Chem. Biol.* 9, 362–366. Copyright (2013), with permission from Macmillan Publishers Ltd.)

Self-assembling polypeptides are created by leveraging natural protein oligomerization domains to form higher-order structures. The interfacing surfaces of these domains can be tuned for assembly into tetrahedral and octahedral structures.^1^ Yeates Group leverages on symmetry in designing novel protein cages.^33^^,34^ Two or more subunits are fused in a geometrically predefined manner which subsequently self-assemble into highly symmetrical structures (Figure 6).^35^ The design principles have led to the production of megadalton protein nanocages with icosahedral symmetry.^36^ Gradišar *et al.*^37^ proposed a different strategy inspired by DNA origami approach. polyhedral structures consisting of individual self-assembling polypeptide nanostructures based on modularized orthogonal dimerizing segments. The formation of these dimerizing segments is controlled by electrostatic and hydrophobic interactions that serve as a platform for designing new artificial polypeptide folds (Figure 7).^37^ The interlinked coiled-coil segment-based design can lead to asymmetric structures that are challenging to construct by the assembly of organic and inorganic subunits. The enormous size of existing protein structural databases could aid the design of a diverse class of self-assembling structures.^35^

### Hybrid protein scaffolds

**Figure 8:**
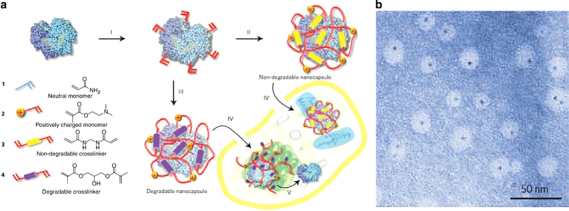
(**a**) Schematic representation of nanocapsule synthesis by *in situ* copolymerization of degradable and non-degradable nanocapsules from their constituent crosslinked subunits (I, II and III), followed by the cellular uptake of the formed nanocapsules via endocytosis (IV); eventually, the loaded protein cargoes are released via shell degradation in the intracellular environment (V). (**b**) TEM image of nanocages containing one 1.4-nm gold quantum dot-labeled horse radish peroxidase core within the nanoscale architecture, scale bar=50 nm^2^ (adapted from Yan, M. *et al.* A novel intracellular protein delivery platform based on single-protein nanocapsules. *Nat. Nanotechnol*. 5, 48–53. Copyright (2010), with permission from Macmillan Publishers Ltd).

**Figure 9:**
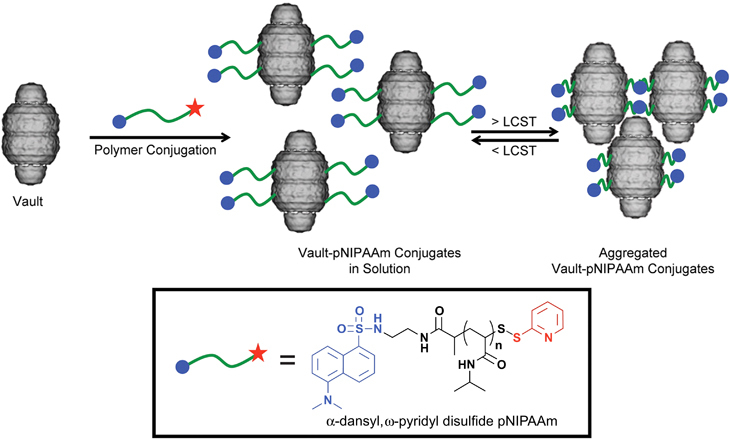
Synthesis of thermoresponsive vault conjugates. The recombinant vault images are cryo-EM reconstructed^28^ (reprinted (adapted) from Matsumoto, N.M. *et al.* Smart vaults: thermally-responsive protein nanocapsules. *ACS Nano* 7, 867–874. Copyright (2013), with permission from American Chemical Society).

To expand their applications in diagnostics/therapeutics and to modulate the immune response, protein nanocages have been conjugated with other moieties (i.e. sugar, lipid, nucleotides and polymer) that result in hybrid protein scaffolds. Synthetic polymer conjugates of viruses, such as tobacco mosaic virus, adenovirus, and cowpea mosaic virus, are the most widely reported hybrid conjugates of protein scaffolds.^38^ Lucon *et al.*^39^ synthesized poly(2-aminoethyl methacrylate) from the interior of P22 viral capsids through atom-transfer radical polymerization, producing particles with increased loading capacity for magnetic resonance imaging (MRI) contrast agents, Gd-DTPA.^39^ Protein nanocages could be enclosed within a polymeric shell attached covalently to the external surface of the protein core.^2^ Polymerizable vinyl groups are grafted to the protein, and further polymerization with crosslinkers and monomers favors the formation of a thin polymer coat around the protein. Such strategies are designed to produce protein nanocages with a non-degradable or degradable polymeric shell for designated purposes (Figure 8).^2^ Similarly, introducing a certain class of ‘smart polymers’ could impart stimuli-responsive properties to protein nanocages.^28^ Smart polymers are designed to be sensitive to external factors such as heat, pH, magnetic field and enzymatic degradation, as well as other microenvironmental changes. For instance, pNIPAAm (poly(*N*-isopropylacrylamide)) is a well-known temperature-sensitive polymer that undergoes a reversible phase change, losing its water solubility above the lower critical solution temperature.^28^ Matsumoto *et al.*^28^ produced novel biohybrid nanoparticles through covalent linkage of pNIPAAm to recombinant CP-MVP (cysteine-rich 12-amino-acid peptide to the N-terminus of the MVP) vaults (Figure 9).^28^ The resulting polymerized particles were thermally responsive as expected. Although synthetic polymer–protein nanocage hybrids have been widely explored, reports on sugar, polysaccharide, nucleotide or lipid modifications are limited.

## Desired properties of nanocarriers in biomedical applications

The properties of carriers for delivery of drugs, contrast agents or other active molecules should be considered depending on the target and specific therapeutic index to be achieved. Certain essential factors include drug efficacy, the target physiological environment, stability, safety, loading capacity and drug release kinetics of the carrier.^40^ Carriers measuring 25–50 nm favor receptor-mediated endocytosis and target cell membrane encapsulation.^8^ An advantage of nanocage-based carriers is the ability to decorate these nanocarriers with multiple functional elements precisely and uniformly.^8^ These functional elements aid the carriers because of their ability to modulate the immune response, their penetration efficiency, and their targeting capability for delivery of active molecules. Most importantly, the carrier should be non-toxic for clinical applications.^40^ Three main properties underlying the design of a drug carrier are discussed in the following subsections.

### Biocompatibility and biodegradability

An ideal drug carrier should have significant biocompatibility. The definition of biocompatibility pertaining to drug delivery systems has been concisely reviewed by Kohane and Langer.^41^ A more comprehensive review by Naahidi *et al.*^42^ emphasized that the ideal drug carrier should be devoid of intolerable toxic, thrombogenic, carcinogenic and immunogenic effects. The carrier should be sustained in the host long enough to carry out its intended function; therefore, the half-life of the carrier is an essential factor for ensuring its effectiveness. The delivery vehicle should be cleared from the biological system after performing its function without settling in any organs, which can lead to long-term side effects. The biocompatibility of a carrier nanoparticle is thus a relative property that depends on the risk–benefit ratio regarding its overall role without causing considerable damage to the body of the host. The suitability of the type of carrier used might vary from one context to another, but the end result should be an appropriate host response.^42^ One of the major concerns associated with protein nanocarriers, which have repeated virus-like structural motifs, is their immunogenicity. Elevated immunoglobulin G levels and B-cell numbers have been noted following a single dose of heat-shock protein and CPMV.^8^ Multiple administrations could thus enhance immune responses and neutralize the carriers. Nanocarriers are cleared when they start to bind to proteins called opsonins, which mediate phagocytosis by the reticuloendothelial system of the host.^42^ A common modality for reducing the immunogenic response would be to modify the carrier surface with poly(ethyleneglycol) (PEG), polymethylmethacrylate, poly(lactic-co-glycolic acid) or polyamidoamine.^3, 42^ Alternative methods include coating with polyketals or glycan shielding.^8^

Biodegradable carriers that are digested and cleaved by the body are often chosen over non-biodegradable ones, for they might cause toxic side effects. Such preferred carriers can be prepared from polysaccharides (e.g. chitosan), proteins, gelatin or biodegradable synthetic polymers such as poly(lactic-co-glycolic acid) and polylactic acid.^42^ As they are made of proteins, protein nanocages are naturally biodegradable.

### Release behavior

One of the key requirements of drug delivery systems is the effective loading and release of drugs from the nanoparticle carriers. The strategies used to choose a delivery system rely on the system’s structure and functions, the nature of the drug carried and the microenvironment the drug encounters. The delivery of drugs/active molecules can be facilitated by chemical immobilization, non-covalent interaction and environment-dependent conformational changes of the carrier. Protein-based carriers and drugs could be chemically conjugated by post-translational attachment of drug molecules to reactive side chains of amino acids such as amines, carboxyls, sulfhydryls and hydroxyls, as well as non-side chains through click chemistry. Covalent conjugation allows for adequate control over release kinetics. The mode of drug release is chosen based on the conjugation chemistry between the drugs, the carrier and the cellular microenvironment encountered. The interior cavity of the protein nanocages often contains natural reactive sites for attachment of nucleic acids, drugs or metals. Drug molecules may also be loaded by nonspecific interactions with secondary carriers that have a strong affinity for the internal surface of the carrier. The nonspecific interactions may modulate drug release kinetics under physiological conditions. Diffusion through the native pores in the carrier facilitates entry into the central cavity. Gated pores in certain VLPs could swell open at low salt concentrations, high pH or osmotic shock, helping load the drug. When the conditions are reversed, the drug is retained, preventing outward diffusion. This mechanism can be applied to deliver drugs to acidic cancer microenvironments. The disassembly and reassembly of the protein cages based on environmental conditions could also aid drug loading and release. The release mechanism of loaded molecules could be tuned by introducing repulsive forces at the intersubunit interfaces to facilitate environment (e.g. pH)-triggered dissociation of protein subunits of the nanocarrier.^8, 43^

### Targeting

Nanoparticles accumulate in the cancer microenvironment by enhanced permeation and retention (EPR) effect due to the leaky vasculature of the tissue. Nonspecific accumulation through the enhanced permeation and retention effect is referred to as passive targeting and may not be very effective. To enhance the affinity for target cells, the carriers can be decorated with targeting ligands to impart active targeting. Targeted delivery of drugs by carrier systems can reduce the amount of drug-carrier complex needed for therapy. An example of active targeting ligand is the peptide RGD, which is present in adenoviruses and has a natural affinity for upregulated integrin receptors in endothelial cells of tumor vessels. Attaching this peptide to the external surface of a carrier aids tumor-targeted delivery. Certain tumor delivery platforms focus on targeting the surface receptors using natural ligands that facilitate endocytosis,^44^ such as folate receptors in cancer cells, transferrin receptors and epidermal growth factor receptors.^40^ Although there are many diseases that delivery systems target, research has focused on solid tumors, and cardiovascular diseases. One of the emerging areas of interest is the modulation of the immune response using protein carriers for application in tumor immunotherapy and the treatment of autoimmune diseases.^8^ Other ligands used for biological targeting include antibodies, engineered antibodies (e.g. single-chain variable fragment) and non-natural peptide ligands. Non-natural peptide ligands are often identified using phage-display libraries that provide the opportunity to screen multiple ligands with different affinities towards a specific cell receptor. Aptamers are nucleotide-based molecules that have been garnering interest as targeting ligands. The targeting ability of a carrier depends mainly on its surface ligand density, which can be tuned on protein nanocages to suit a given application.^8, 45^

## Engineering of protein scaffolds for specific biomedical applications

Similar to other nanoparticles, the application of protein nanocages in biomedicine involves several challenges: (1) lack of a natural capability to carry drugs, (2) lack of specificity, (3) low cell uptake efficiency, (4) absence of endosomal escape mechanism, (5) limited circulation time, (6) potential to trigger an immunological response and (7) lack of tuneable release properties. Furthermore, clinical applications of nanocages are limited because of the structures’ poor stability and cell permeability.^2^ To overcome some of these challenges, the engineering of protein nanocages is required to impart non-natural functions. For example, the translocation of these carriers into cells through endocytosis is not always advantageous as these materials are digested in the lysosome rather than shuttled into the target cell organelle. Hence, incorporating an endosomal escape mechanism into protein nanocages is beneficial. What distinguishes nanocage-based delivery systems from other inorganic nanoparticles is the spatial control of functional groups and the ligands attached to the protein structure. Multifunctional properties can be achieved by combining the desired modifications for loading, targeting and chimeric assembly, leading to the development of smart nanocarriers with tremendous potential in nanomedicine.^4^ Selective covalent chemical modifications in protein nanocages are made by leveraging native amino acids such as lysine, glutamic acid, aspartic acid and cysteine.^7^ Table 1 provides a library of natural protein cage structures that have been chemically or genetically engineered to impart functional groups to different cage surfaces, such as the interior, exterior or intersubunit interface.

**Table 1:** Modification of protein nanocages, tags, effects and applications

*Protein cage type*	*Modification procedure*	*Engineered surface/interface*	*Architectonic tags*	*Effects*	*Applications*	*Refs.*
HAdv-5	Genetic engineering	Capsid protein fiber	HAdV-3 capsid protein fiber head domain instead of HAdV-5 head domain	Altered viral tropism, switch in receptor specificity, increase in binding affinity and entry into cells	Transduction into primary melanoma cells	^46, 47^
CPMV	Chemical and genetic modification	Viral cage surface	Fluorescent dyes, stilbene derivatives, polyethylene glycol chains, biotin, DNA, peptides such as neuropeptide Y, carbohydrates and antibodies	Enhance binding affinity to target receptors and reactive amino acids, regioselective targeting	Anti-viral and antitumor applications	^3^
CCMV	Chemical modification	Viral cage exterior surface	Fluorescent molecules and small peptides (reaction with surface-exposed lysines, sulfhydryls, carboxylic acids and using copper-catalyzed alkyne azide ‘click’ chemistry)	Site-specific attachment of target ligands, multiligand display on the outer surface, attachment to large polymeric species	Material and biomedical applications such as inducing protecting immunity, which can lead to vaccine development	^3, 48^
TMV	Genetic engineering/recombinant technology and chemical conjugation	Viral coat exterior and interior surface	Biotin, chromophores and crown ethers, PEG polymer, immunogenic peptides, for example, malaria B-cell epitope	Altering the solubility of viral particles, display of biological ligands, uptake and activation of immune cells, inducing antibodies	Preparation of linear arrays of chromophores, inorganic nanoparticles and multicomponent materials Development of vaccines/injectable biologics.	^49, 50^
Bacteriophage MS2	Bioconjugation/chemical modification	Viral capsid exterior and interior surface	Targeting ligands, florescence dye molecules, PEG polymer decorated on the exterior, biotin, small-molecule targeting groups	Cell-specific uptake, degradable interior linkage for drug cargo, no disruption in the self-assembly of the cage proteins	Targeted drug delivery and imaging	^51, 52^
Bacteriophage M13	Genetic modification and chemical conjugation	Viral coat protein	αv-Integrin-binding peptide, streptavidin-binding adaptor moiety	Bifunctional attachment of molecules for cell specificity and delivery of imaging agents	Phage display, tumor targeting, drug delivery, imaging, luminescent quantum dots localization, surface plasmon resonance-based binding detection and templating inorganic materials	^52, 53^
TYMV	Chemical and genetic modification	Exterior coat protein	Short-chain polymers, epitope peptides, RGD-containing peptide targeting moieties	Control of virion assembly and RNA packaging, improved cell adhesion, cell proliferation and spreading	Tissue engineering, drug delivery, and biosensing	^54, 55^
*Methanococcus jannaschii,* Hsps	Chemical and genetic modification	Exterior and interior surface	Tumor vasculature targeting peptides, for example, α–β-integrin, antibodies such as anti-CD4 antibodies and fluorescein molecules	Cellular tropism, multifunctional behavior, cell-specific targeting, cellular uptake, immune modulation	Delivery of imaging and therapeutic agents, oxidative mineralization of iron and synthesis of organic and inorganic molecules	^56, 57^
Bacteriophage MS2-derived virus-like particles	Chemical bioconjugation	Interior surface	miRNA, Tat peptide,	Targeted molecular anchoring, aid in cell penetration behavior and target gene suppression	Gene function analysis and gene therapy	^58^
Rotavirus structural protein-derived virus-like particles	Chemical conjugation	Interior surface	Lactobionic acid	Multifunctional behavior and specific targeted delivery	Antitumor therapy and other biomedical applications	^59^
Cowpea mosaic virus-based viral-like particles	Chemical conjugation	Exterior surface	Anticarcinoembryonic antigen	Specific targeted delivery to HT-29 human tumor cells	Antitumor therapy and other biomedical applications	^60^
Polyomavirus-like particles	Genetic modification	Exterior capsid protein	WW domain fusion sequence(for binding to proline-rich ligands)	Short-term coupling of external ligands, capsid assembling efficiency similar to the wild-type viral particles	Drug delivery applications	^61^
M13 bacteriophage virus-like particles	Genetic modification	Exterior capsid protein	αv-Integrin binding peptide, streptavidin-binding adaptor moiety	Bifunctional ligand display, simultaneous delivery of targeting and imaging agents	Drug delivery and imaging, Antitumor therapy	^9, 60^
*Aquifex aeolicus* lumazine synthase bacterial microcompartments	Genetic and chemical modification	Interior surface	Green fluorescent protein, positively charged amino acids, introduction of glutamate residues	Encapsulation of active molecules of interest by charge complementarity	Delivery of drugs and imaging agents	^62^
Propionaldehyde dehydrogenase bacterial microcompartments	Genetic modification	Exterior and interior surface	Green fluorescent protein, glutathione *S*-transferase, RGD-4C peptide, anti CD4 monoclonal antibody	Encapsulation of active molecules, targeting specific cell environment of organs, tissues and tumors	Delivery of drugs and imaging agents, antitumor therapy.	^63^
Ferritin	Chemical and genetic modification	Interior and exterior surface	Interior—Iron nanoparticles, metal ions, palladium particles, gold, rhodium norbornadiene, ferromagnetic/antiferromagnetic nanoparticles	Nanoparticle formation, modifying magnetic properties of nanoparticles, magnetic bias behavior, MRI contrast agent formation, catalytic activity, tumor targeting, and cell binding affinity	Nanoparticle synthesis, high-density recoding, sensors, mineral synthesis, MRI, polymer synthesis, tumor therapy, drug delivery vehicle	^18, 64, 65^
			Exterior—RGD−4C peptide, integrins, epitopes/antigens.			
DNA-binding proteins from nutrient-starved cells	Chemical and genetic modifications	Interior and exterior surfaces	Interior—Metal-binding ligands	Metal core formation, toposelective modification, polarized orientation of functional groups, hierarchical assembly of subunits.	Imaging and drug delivery vehicles	^64^
			Exterior—fluorophores, streptavidin, targeting peptides, epitopes			
Eukaryotic vaults	Chemical and genetic modifications	Interior and exterior surface	Exterior—Cysteine-rich amino-acid peptide, epitopes, IgG-binding peptide and epidermal growth factor	Cell-specific targeting of epithelial cancer cells, increasing particle stability, controlled release of drugs	Imaging and drug delivery vehicles	^27, 66, 67^
			Interior—proteins, nanoparticles, gold probes and polymers			
*Geobacillus stearothermophilus* E2 protein	Chemical and genetic modifications	Interior, exterior and inter subunit interface	Interior—cysteines, fluorescent dye and drug molecules, CpG DNA motif	pH-controlled assembly, induction of antibodies, drug loading, cell-specific targeting similar to folate receptor targeting in cancer cells	Imaging and drug delivery vehicles	^22, 43, 68^
			Subunit interface—histidine residues			
			Exterior—antigenic epitopes, folate, targeting ligands, EGFR antibodies			
Inclusion bodies	Genetic modification	Interior cage surface	Green fluorescent protein, β-galactosidase, β-lactamase, alkaline phosphatase, D-amino-acid oxidase, polyphosphate kinase 3, maltodextrin phosphorylase sialic acid, aldolase	Encapsulation of peptides by the inclusion body protein scaffolds followed by protein aggregation	Protein expression, purification, industrial biocatalysis, drug delivery devices for bionanomedicine	^1, 69^

Abbreviations: CCMV, cowpea chlorotic mottle virus; CPMV, cowpea mosaic virus; HAdv-5, human adenovirus type-5; Hsps, heat-shock proteins; IgG, immunoglobulin G; PEG, poly(ethyleneglycol); TMV, tobacco mosaic virus; TYMV, turnip yellow mosaic virus coat protein.

### Sites of engineering

**Figure 10:**
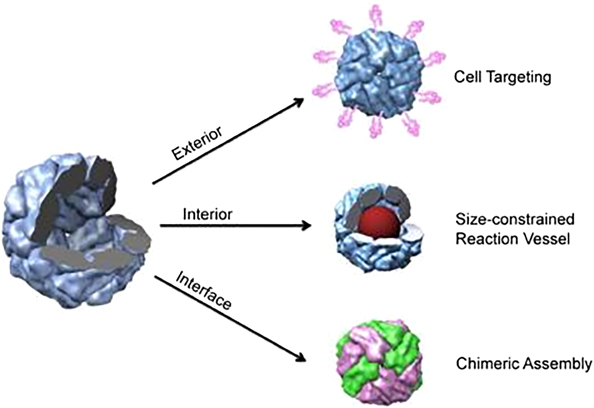
Protein cage modifications at distinct interfaces^64^ (reprinted from Uchida, M., Kang, S., Reichhardt, C., Harlen, K. & Douglas, T. The ferritin superfamily: supramolecular templates for materials synthesis/ferritin: structures, properties and applications. *Biochim. Biophys. Acta* 1800(8), 834–845. Copyright (2010), with permission from Elsevier).

The structure of protein nanocages offers three distinct surfaces for engineering: the interior, exterior and intersubunit interface (Figure 10).^64^ These interfaces can be exploited to induce chemical and genetic modifications for various purposes.^64^ These modifications can also be made under the same reaction conditions, providing the opportunity to control the size, position and orientation of the loaded external active molecules.

#### Interior modification of protein nanocages

**Figure 11:**
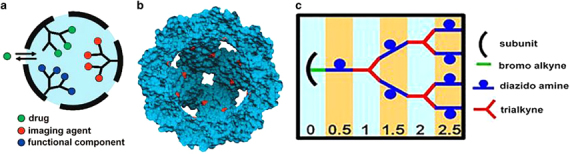
Design of protein nanocage-polymer hybrids. (**a**) Representation of protein cage with polymer branches in the interior cavity with attachment sites for drugs or imaging agents. (**b**) A cross-sectional view of HSPG41C nanocage showing cysteine residues (red) in the interior cavity. (**c**) Scheme for the production of dendritic structure with the generation units indicated at the bottom^71^ (reprinted (adapted) from Abedin, M.J. *et al.* Synthesis of a cross-linked branched polymer network in the interior of a protein cage. *J. Am. Chem. Soc.* 131, 4346–4354. Copyright (2009), with permission from American Chemical Society).

The interior cavity of the protein nanocages provides an ideal containment for molecular cargos. Tailoring the cage interior increases the encapsulation efficiency, binding affinity and modulated release profile. Genetic or chemical alterations can be made at precise locations to manipulate the nucleation and attachment of molecules. Molecular cargos such as small molecules, peptides, protein drugs, RNA/DNA drugs, imaging agents and polymers have been encapsulated and released from the interior of protein nanocages. Antibodies are potential cargo for larger protein nanocages, such as vaults. Modification of the nanocage interior by introducing cysteine residues has been a widely used approach. The modification facilitates covalent attachment of dye molecules, drugs and other active molecules through disulfide bonds.^5^ Other modifications include the introduction of phenylalanine and the attachment of lipid substances and polymers inside the cage architecture.^70^ The attachment of polymer chains to the interior surface offers spatial control of reactive sites, which has been shown to stabilize cages. Abedin *et al.*^71^ created a polymer network inside a protein cage by introducing cysteine reactive residues by genetic manipulation. The polymer network was created by the sequential conjugation of multifunctional monomeric units by click chemistry, allowing the free amines to be incorporated into the polymer for internal functionalization (Figure 11). The polymer network covalently crosslinks the subunits of the protein cage, increasing its thermal stability to at least 120 °C.^71^

#### Exterior modification of protein nanocages

The exterior surface of protein nanocages is engineered to impart increased circulatory half-life, local accumulation, cellular penetration and triggering of specific cellular responses. The unique geometry of protein nanocages allows for multiple ligands and functional molecules to be displayed on their surfaces. Modifying molecules include small molecules (e.g. folate), peptides (e.g. RGD, transactivator of transcription 47–57 peptide (cell penetrating peptide), immunoglobulin G binding peptide (Z domain)), antibodies (e.g. anti-epidermal growth factor receptor and anti-CD4), nucleotides (e.g. DNA, RNA, aptamers) and polymers (e.g. PEG, PGA, poly(lactic-co-glycolic acid), polymethacrylate, polyamidoamine). The exterior surfaces of CPMV, cowpea chlorotic mottle virus (CCMV) and bacteriophages M13, Qb and MS2 have been modified with antibodies, peptides, transferrin and cell transduction domains or cell-penetrating peptides.^58^ The adenovirus-based peptide RGD has a natural affinity for integrin receptors that are upregulated in tumor vessels. Modification of protein carriers with this peptide has been proven to aid tumor targeting.^8^ Short, single-stranded oligonucleotides called aptamers with secondary structures capable of recognizing specific molecules can be incorporated onto the nanoparticles to enhance their specificity. These aptamers can bind to both extracellular (membrane) and intracellular proteins, thus facilitating aptamer–nanoparticle conjugate therapy.^72^ Other functionalities can be imparted to the nanocages by attaching functional ligands to extend their half-life (e.g. PEG) and enhance penetration into cells (e.g. cell penetrating peptides). Folate-PEG displayed on adenoviral nanoparticles has been delivered to folate receptor-overexpressing cells. These nanocarriers were proven to decrease the interleukin-6 levels of macrophages, showing that the engineered viral scaffolds counteract the innate immune response.^1^ PEG acts as a stealth layer shielding the immunogenic epitopes on the surface of the nanocage structure.^73^ This layer prevents the protein opsonin from adsorbing onto the surface, thereby shunting the recognition of the nanocage by phagocytic cells (reticuloendothelial system).^42^ The PEG modification also benefits the tuneable solubility and structural integrity of the protein nanocage.^73^

The spatial control of multifunctional groups on protein nanocages equips them for both hierarchical self-assembly and display of distinct functional ligands. Display of multiple types of ligands in a precise arrangement on nanoparticles is challenging. A distinct advantage of protein nanocages over other nanoparticles is that the position of each amino acid is spatially defined, allowing for precise spatial control of the displayed ligands. The N or C termini of the nanocage subunits that face the external surface are a natural choice for displaying functional ligands. Fusion of short peptides to these termini has been achieved through genetic engineering. Modification of recombinant vault by fusing a cysteine-rich 12-amino-acid peptide to the N terminus of the MVP (CP-MVP) leads to increased particle stability.^67^ Engineering vault MVP C-terminal regions with tags such as epitopes, 33-amino-acid immunoglobulin G-binding peptide (Z domain) or 55-amino-acid epidermal growth factors, which are displayed at the caps of the vault, were shown to facilitate cell-specific targeting.^27^ In another approach, Domingo *et al.*^74^ denatured, mixed and reassembled protein subunits carrying different chimeric peptides to display multiple types of ligands (i.e. green fluorescent protein and two types of malaria epitopes) on the surface of a single E2 protein nanocage. The display of two types of HIV-derived antigenic epitopes (pep23 and RT2) provokes specific antibodies and T-cell responses.^74^

**Figure 12:**
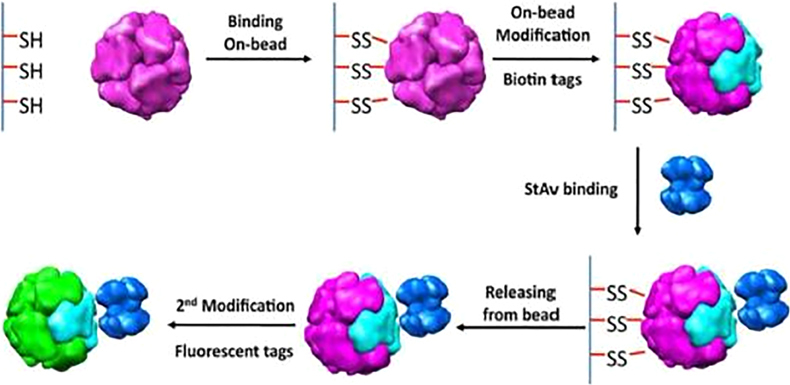
Toposelective modification of *Listeria innocua* DNA-binding protein nanocage using masking/unmasking method using a solid bead support^64^ (reprinted from Uchida, M., Kang, S., Reichhardt, C., Harlen, K. & Douglas, T. The ferritin superfamily: supramolecular templates for materials synthesis/ferritin: structures, properties and applications. *Biochim. Biophys. Acta* 1800(8), 834–845. Copyright (2010), with permission from Elsevier).

Despite the natural spatial control in protein nanocages, achieving the desired symmetry is still a great challenge.^64^ Production of protein nanocages with a dual architecture was achieved by toposelectively modifying the exterior surfaces of DNA-binding protein from starved cell cages by a masking/unmasking method based on solid supports. The distribution of the two functional domains, fluorophore and affinity tag, was manipulated by using different materials for the solid supports (Figure 12).^75^ This toposelective modification provides more sophisticated designs of multifunctional nanoplatforms. By combining genetic fusion with toposelective attachment of streptavidin, the universal coupling protein to which a biotinylated molecule could be attached, Suci *et al.*^76^ achieved asymmetrical placement of streptavidin. This asymmetrical placement provides further spatial control over the display, providing tools with which to explore the effects of the polarized orientation of conjugated functional molecules on interactions with specific cell surface receptors.

#### Intersubunit modifications

The self-assembly of nanostructures in cells depends on specific protein–protein interactions. Understanding these interactions is essential in the design of novel nanostructures for various applications. For example, controlling the assembly and disassembly process to respond to pH variations has implications for the release of molecular cargos from the protein nanocage. Other triggers include salt concentration and nucleic acid content, as well as metal ions.^5^ The display of ligands can also be modulated by modifying how the subunits interact.

**Figure 13:**
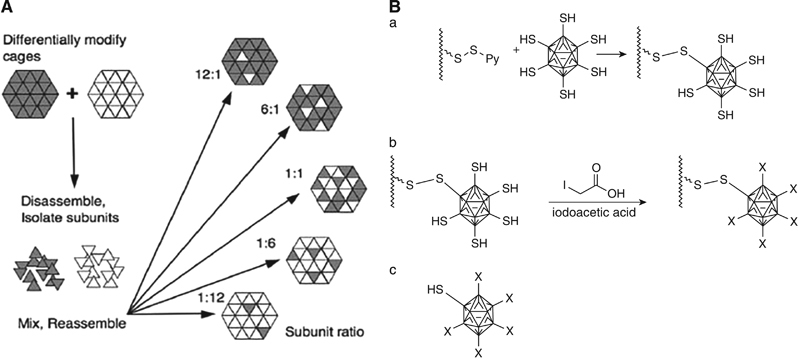
Different methods for asymmetric ligand display on cowpea chlorotic mottle virus (CCMV) nanocage. (**A**) Dissociation of differentially engineered CCMV cages into subunits and reassembly. (**B**) Symmetrical dissociation of a CCMV mutant A163C. (**B**a) Thiol modification of the viral nanocage by activated resin. (**B**b) Neutralizing the unbound cysteines using IAA (**B**c) Separation of symmetry broken mutant nanocage reduction^5^ (reprinted from Uchida, M., Klem, M.T., Allen, M., Suci, P., Flenniken, M., Gillitzer, E. *et al.* Biological containers: protein cages as multifunctional nanoplatforms. *Adv. Mater.* 19, 1025–1042. Copyright (2007), with permission from John Wiley and Sons).

The interface between subunits of viruses such as CCMV was studied to determine the mechanism of supramolecular assembly of protein nanocages.^77^ In-depth study of the interactions between protein subunits at the interface has paved the way to optimizing the conditions for the induction of self-assembly. Genetic modification of specific locations on the subunits, especially at the N and C termini, yields VLPs with different sizes and total number of subunits.^77^ The CCMV viral cage was also tested in terms of low symmetrical assembly. Two different strategies were developed to break the symmetry of ligand presentation on the virus.^5^ CCMV nanoparticles were labeled differently with either biotin or digoxigenin and then were disassembled into individual subunits (Figure 13). These differentially labeled subunits were purified, mixed and reassembled in optimal stoichiometry to form dual-function nanocages with controlled display of ligands. The maximum benefit of this strategy can be reaped when a functional nanocage structure is assembled based on a library of differentially engineered subunits, each having its own ligand or epitope.^5^

Protein–protein interactions often involve large areas of buried surface that can be interrupted by small molecules that target ‘hot spots’ where the energy for binding is concentrated.^78^ Zhang *et al.*^78^ identified these hot spots in DNA-binding protein from starved cell by the alanine shaving method. This method works by conceptually shaving the individual side chains to methyl residues; the stabilities of the resulting mutants are deduced by comparing them with the wild type. This work could aid the basic understanding of the assembly of miniferritin and the control of ferritin’s oligomerization state, which can be applied as templates for nanomaterial synthesis and drug delivery.

By modifying the interactions at the subunit–subunit interface, the assembly profile of E2 protein nanocages can be modified such that they are stable at pH 7.4 but disintegrate at acidic pH.^43, 79^ In other words, the pH transition point at which this change occurs can be controlled. Using this strategy, the release of the encapsulated drug/active molecules can be achieved by modulating the interactions between the subunits to respond to pH changes in the target cell microenvironment.^79^ For example, the tumor microenvironment is slightly more acidic than healthy tissues. pH changes are also experienced by molecules entering cells by endocytosis. The molecules will experience a pH level of 7 near the cells and then a pH level of 5 upon entering the lysosome later in the pathway. Thus, pH-based assembly and disassembly responds could be used to trigger the release of molecules encapsulated within protein nanocages.

#### Engineering and production of protein nanocages

Protein nanocages are plastic and powerful materials because of their low toxicity, functional diversity and flexibility in being suitably engineered for specific purposes.^6^ The construction of protein nanocages from their building blocks could be achieved by selecting protein domains that facilitate protein–protein interactions without nonspecific aggregation. Environmental parameters such as temperature and pH have been used to control peptide self-assembly.^6^ Additional functional ligands can be incorporated into naturally existing protein nanocages by genetic engineering and chemical modification techniques. Genetic engineering of the protein nanocages is achieved by recombinant DNA technologies. They are produced in small and large scales using both microbial and non-microbial systems as the cell factories. Bacterial cells, in particular, are popular hosts for producing a variety nanomaterials, including those for medical applications.^6^ Chemical modifications can be used to prevent the interbatch variability of protein production observed in recombinant DNA technologies.^1^ The Francis group has developed new methods for chemically modifying proteins that may be applicable to protein nanocages.^80^

### Application of protein nanocages as drug/gene carrier

By leveraging on facile genetic or chemical modifications to promote molecular attachment and encapsulation, protein nanocages can be engineered to carry drugs, genes, imaging contrast agents or other active molecules. To date, small molecules such as doxorubicin, bleomycin, cisplatin, carboplatin, nucleic acids DNA/RNAs, peptides and proteins such as Cre recombinase, capase-8, interferon-γ and interleukin-2 have been delivered *in vitro*, whereas paclitaxel, doxorubicin, interleukin-2, CD40L, small interfering RNA, microRNA and CpG oligos have been applied *in vivo* as therapeutics using protein nanocages.^8^

Covalent attachment of small molecules to the interior of protein nanocages prevents leakage, protects the drugs by preventing degradation and prevents nonspecific interaction. For instance, the low endosomal pH of the cell could be used as a trigger to release the encapsulated drug. The heat-shock protein cage interior and E2 protein are modified by genetically inserting cysteine residues at the interior surface for selective attachment of antitumor drugs with a pH-sensitive linker.^68, 81^ The attachment of 6-maleimidocaprol, a hydrazone derivative of doxorubicin, to the inner surface of cysteine-modified heat-shock protein and E2 protein nanocages allows for the selective release of the drug under acidic conditions through the hydrolysis of the hydrazone linkage. Ferritin has been used to encapsulate the platinum-based anticancer drugs cisplatin and carboplatin.^82^ Drugs encapsulated within a protein nanocage have been reported to retain their efficacy and exert cytotoxic effects on cancer cells. However, efficacy comparisons between the encapsulated drugs and free drugs and their effects on healthy cells are still under investigations.

VLPs such as the brome mosaic virus, CCMV, papillomavirus and polyomavirus have been used to package and deliver exogenous DNAs.^83^ Pan *et al.*^1^ used bacteriophage MS2 VLPs to deliver human pre-miRNA. The nanocages were simultaneously decorated with cell-penetrating peptides and transactivator of transcription 47–57 to enhance cellular penetration. The designed delivery vehicle promotes subcellular localization of nucleic acid miR-146a, which suppresses the target gene effectively.^1^ Small interfering RNA encapsulated in HBV capsid nanocarriers was shown to systemically deliver small interfering RNA to cancer sites in mice and effectively silences the target gene.^84^

Protein nanocages have also been explored as carriers for protein-based therapeutics. Delivery of protein therapeutics requires protein nanocages with larger cavities. Model therapeutic molecules, such as prodrugs and enzymes, have been packaged in VLPs by fusing them to Gag proteins.^85^ Viral vectors are naturally taken up by cells through the interaction of the envelope protein on their outer surface with cell receptors.^1^ VSV-G envelope^86^ or other localizing targeting ligands further increase the efficiency of delivery of the cargos to particular cells. Vaults with a volume of ~122,000 nm^3^ are good non-viral protein nanocages candidates for packaging of protein-based therapeutics^26^. Loading can be facilitated by fusing the protein of interest with INT, which serves as a shuttle protein to package the molecule inside the vault.^27^ Modifications of the vault interior and exterior to accommodate other therapeutics or other ligands are reviewed in other sections.

### Application of protein nanocages as imaging agents

The basic requirements for *in vitro* and *in vivo* imaging is to achieve high local concentrations of imaging agents and suppression of the quenching of fluorescent probes. Various contrast agents for enhanced MRI, positron electron tomography and near infrared fluorescence imaging have been encapsulated in protein nanocages. Single or coencapsulation of the contrast agents will allow for the development of protein nanocages with multimodal imaging ability. Of all protein nanocages, ferritin has dominated bioimaging applications. The inherent ability of ferritins to store iron as ferric oxyhydroxide particles makes them attractive as an MRI contrast agent. The ferric oxyhydroxide nanoparticles are superparamagnetic; therefore, endogenous ferritin loaded with these particles can act as a natural T2 MRI contrast agent.^87^ At clinically significant magnetic field strengths (i.e. 1.5 and 3 T), ferritin has 10–100-fold less relaxivity per iron than commercially produced iron oxide nanoparticles. Loading of superparamagnetic iron oxide nanoparticles in ferritin nanocages shows higher *r*_2_ relaxivity than endogenous ferritin.^64^

Protein nanocages can be engineered in a chemoselective manner to increase the loading capacity of fluorophores (e.g. Alexa Fluor or fluorescein dyes on CPMV) with precisely defined positions on the exterior and interior of the cage. The imaging agents are attached to the rigid and ordered protein structure at distinct positions. The spatial confinement of dye molecules prevents them from reacting or aggregating with each other and therefore reduces the quenching possibility. The inherent ability of some near-infrared fluorescence dyes to penetrate tissues with reduced background noise makes them attractive for *in vivo* imaging.^3^ Attachment of these dyes on the protein nanocages increases the local concentration resulting in reduced dosing. A notable example is luminescent semiconductor nanocrystals (quantum dots), which were displayed on engineered CPMV nanocages.^3^ Bacteriophage MS2 nanocages were tested for their ability to carry the positron electron tomography imaging agent [^18^F]fluorobenzaldehyde. The radiolabel was attached to the interior of the cage by bioconjugation. Positron electron tomography imaging analysis in rats showed that conjugation with protein nanocages increases the blood circulation time of the imaging agent without affecting biodistribution.^88^ Protein nanocages can also be used as contrast agents in ultrasound imaging. Gas-filled protein based nanostructures derived from cyanobacterium *Anabaena flos-aquae* has been genetically engineered for enhanced harmonic properties that is optimal for *in vitro* and *in vivo* ultrasound imaging.^87^ It has been shown that these protein nanocages can also be engineered for multimodal and targeted ultrasound imaging.

As each imaging modality has unique advantages such as tissue penetration depth, spatial and temporal resolution and cell specificity, the combination of multiple molecular probes of different imaging modalities will allow for more accurate diagnosis of a disease condition. To date, only near-infrared fluorescence-positron electron tomography multimodal imaging using protein nanocages has been reported, in which ferritin is used as a carrier to deliver the two imaging probes Cy5.5 and ^64^Cu and a targeting ligand for imaging tumors.^65^

### Application of protein nanocages as vaccine/immune modulators

**Figure 14:**
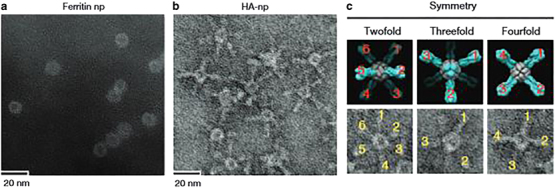
TEM image of ferritin nanoparticles (**a**), hemagglutinin (HA) ligands displayed on ferritin nanocages (**b**) and comparison (**c**) of symmetry between computational models and TEM images of the engineered octahedral nanoparticles displaying HA spikes (numbered)^91^ (reprinted from Kanekiyo, M. *et al.* Self-assembling influenza nanoparticle vaccines elicit broadly neutralizing H1N1 antibodies. *Nature* 499, 102–106. Copyright (2013), with permission from Macmillan Publishers Ltd.).

Protein nanocages have shown promising potential as a display platform for pathogenic epitopes to elicit the production of neutralizing antibodies. HBV, HPV, influenza, HIV, hepatitis C virus, RSV, chlamydia infections and cancers such as cervical cancer and P815 tumors are a few diseases that have been targeted for vaccine development using the protein nanocage platform.^90, 91, 92, 93^ Current vaccines are mostly based on whole viruses, both live attenuated and inactivated. In 1976, Edward Jenner presented the first virus-based vaccine against small pox. Since then, other vaccines have entered the market, including vaccines for hepatitis A, rubella, measles, mumps and influenza. Despite their initial success, the live attenuated viral vaccines are relatively less stable and difficult to administer. There is also the risk that these attenuated viruses will revert to their pathogenic form, which can be detrimental to the host. Neutralized virus vaccines do not carry the risk of reversion, but they are weaker, expensive and exhibit reduced vaccine coverage in a given population. To overcome this problem, subunit vaccines have been considered because they prime the immune response by the administration of a preload of viral proteins, which are often taken from the viral capsid portion. Because there is no viral replication, these vaccines are safer but are less immunogenic when produced and purified without any other viral counterparts. However, the innate ability of viral capsid subunits to assemble into VLPs could be used to mimic the whole virus to improve vaccine efficacy. VLPs could also be used as a platform for displaying foreign ligands of viral or non-viral origins.^8, 92^ Both enveloped and non-enveloped VLP-based vaccines have been developed. Two of the most successful non-enveloped VLP-based vaccines, namely HBV and HPV vaccines, are licensed and commercialized for clinical applications. Viruses such as influenza, hepatitis C virus and HIV can give rise to enveloped VLPs, which lack the ability to replicate. The immunogen is composed of assembled particles consisting of some or all of the surface subunits of the plasma membrane.^92^ Current research is focused on the conserved epitopes of the envelope protein of HIV-1 gp-41. Developing successful HIV vaccine requires engineering different VLPs. The development of VLP-based vaccines against hepatitis C virus, filoviruses Ebola virus, Marburg virus, severe acute respiratory syndrome, coronavirus and chikungunya virus is rapidly progressing.^92, 94^ Plant viruses are also used as a platform for displaying antigenic epitopes. CPMV is an extensively studied plant virus for vaccine applications. Other plant viruses include CMV and PapMV. Bacteriophage and insect virus platforms are also explored for heterologous epitope display.^92^ A variety of cancer vaccines could be developed by using Toll-like receptor agonist CpG peptide-loaded protein nanocages.^8^ Vaccines against respiratory infections, such as influenza and respiratory syncytial virus, have been explored. H1N1-virus specific hemagglutinin was fused genetically to ferritin subunit such that eight trimeric viral spikes were presented on the outer surface after the self-assembly of the nanocage (Figure 14).^91^ Respiratory syncytial viral epitopes have been displayed on VLPs such as baculovirus and Newcastle disease virus.^95, 96^ *Chlamydia trachomatis* immunogenic epitope PmpG-1-loaded vault nanocages, vaginally injected in mice, stimulated specific T-cell responses against the pathogen.^97^ The engineered protein nanocage-based vaccines have been shown to induce protective immunity.

## Concluding remarks and future prospects

Protein nanocages are a versatile platform for biomedical applications. The main advantage of protein nanocages is the spatial control of functional groups displayed at well-defined locations through genetic or chemical modifications. To date, only a few nanoplatforms provide for the ability to simultaneously tune size, shape and biocompatibility. Protein nanocages have been shown to be amenable to engineering to cater to specific functions. Despite the multitude of functionalities, the detailed mechanisms for the uptake of the protein nanocages and their intracellular fates are not fully understood. Although cells internalize some of the carrier molecules, the efficiency of delivery to the intended intracellular compartment remains a challenge. Decorating protein nanocages with ligands, such as cell-penetrating peptides, appears to improve the delivery of drug cargos into the cells via nonspecific cellular uptake. To improve the selectivity and enhance local accumulation on target cells, targeting agents have been displayed on the external surface of protein scaffolds.

The advantage of the spatial control of ligand attachment on protein nanocages, however, is limited with respect to the display of different types of ligands on the same nanocage. Engineering peptides and proteins could have a crucial role in creating multifunctional materials by combining two or more distinct ligands on a single protein nanocage. Selective display of peptides, proteins or nucleotides with such spatial control may open a new avenue for the design and development of novel self-assembling functional hierarchical supramolecular structures. Whereas a similar approach is being pursued in the general field of nanotechnology and still remains a challenge, protein nanocages may present new opportunities.

Although the majority of efforts have been focused on using protein nanocages to deliver anticancer drugs, exploiting protein nanocages to modulate the immune response is an emerging area of research and potentially the most attractive application. Protein nanocages are good candidates for delivering immune modulating agents for application in cancer immunotherapies or autoimmune disease treatments. In nature, viruses have the innate ability to condense and deliver nucleic acids through cell receptor interactions. By studying the structure and functions of the protein subunits in viruses that are responsible for packaging and releasing nucleic acids, novel gene delivery systems can be constructed *de novo*. Other limited explorations include the use of protein nanocages for the treatment of skin conditions such as pigmentation disorders and ocular delivery of therapeutics.

Further development of the vast applications of protein nanocages requires a deeper understanding of fundamental concepts that remain understudied. For example, the self-assembly mechanism of many protein nanocages remains elusive. A detailed mechanistic understanding is important to better design drug loading and release properties. The mechanism of internalization and intracellular processing of these nanoparticles is yet to be determined. Although cell-specific delivery of protein nanocages is possible, the targeting efficiency of these carriers presents a major challenge. In addition, penetration through other physiological barriers such as the epidermal skin layer and corneal layer has yet to be addressed.

The advent of synthetic biomaterials such as artificial peptides, modular proteins and polymers widens the scope for additional properties such as reversible aggregation, which can complement natural scaffolds. The design of hybrid nanoscaffolds *de novo* via nature’s bottom-up approach using smart synthetic materials could provide critical advantages, such as biocompatibility, site-specific modification, control of self-assembly with respect to environmental stimulus, stability and drug/nucleic acid loading. These novel bioinspired materials, which are formed by the self-assembly of distinctly engineered protein subunits for various functions, are of immense value in nanomedicine and associated medical fields.

## Data Availability

GenBank/EMBL/DDBJ
AF158255

## References

[1] Ferrer-Miralles N, Rodríguez-Carmona E, Corchero JL, García-Fruitós E, Vázquez E, Villaverde A (2013). Engineering protein self-assembling in protein-based nanomedicines for drug delivery and gene therapy. Crit. Rev. Biotechnol..

[2] Yan M, Du J, Gu Z, Liang M, Hu Y, Zhang W, Priceman S, Wu L, Hong Z, Zhou H, Liu Z, Segura T, Tang Y, Lu Y (2010). A novel intracellular protein delivery platform based on single-protein nanocapsules. Nat. Nanotechnol..

[3] Lee LA, Wang Q (2006). Adaptations of nanoscale viruses and other protein cages for medical applications. Nanomedicine.

[4] Flenniken ML, Uchida M, Liepold LO, Kang S, Young MJ, Douglas T (2009). A library of protein cage architectures as nanomaterials. Curr. Top. Microbiol..

[5] Uchida M, Klem MT, Allen M, Suci P, Flenniken M, Gillitzer E, Carpness Z, Liepold LO, Young M, Douglas T (2007). Biological containers: protein cages as multifunctional nanoplatforms. Adv. Mater..

[6] Vazquez E, Villaverde A (2010). Engineering building blocks for self-assembling protein nanoparticles. Microb. Cell Fact.

[7] Schoonen L, van Hest JC (2014). Functionalization of protein-based nanocages for drug delivery applications. Nanoscale.

[8] Molino NM, Wang SW (2014). Caged protein nanoparticles for drug delivery. Curr. Opin. Biotechnol..

[9] Douglas, T., Allen, M., Young, M., Fahnestock, S. R. & Steinbüchel, A. Self-assembling protein cage systems and applications in nanotechnology (2005).

[10] Fontana J, Dressick WJ, Phelps J, Johnson JE, Rendell RW, Sampson T, Ratna BR, Soto CM (2014). Virus-templated plasmonic nanoclusters with icosahedral symmetry via directed self-assembly. Small.

[11] Barnhill HN, Claudel-Gillet S, Ziessel R, Charbonniere LJ, Wang Q (2007). Prototype protein assembly as scaffold for time-resolved fluoroimmuno assays. J. Am. Chem. Soc..

[12] Fankuchen I, Ferritin: V (1943). X-ray diffraction data on ferritin and apoferritin. J. Biol. Chem..

[13] Ford GC, Harrison PM, Rice DW, Smith JM, Treffry A, White JL, Yariv J (1984). Ferritin: design and formation of an iron-storage molecule. Philos. Trans. R. Soc. Lond. Ser. B.

[14] Lawson DM, Artymiuk PJ, Yewdall SJ, Smith JM, Livingstone JC, Treffry A, Luzzago A, Levi S, Arosio P, Cesareni G, Thomas CD, Shaw WV, Harrison PM (1991). Solving the structure of human H ferritin by genetically engineering intermolecular crystal contacts. Nature.

[15] Johnson E, Cascio D, Sawaya MR, Gingery M, Schroder I (2005). Crystal structures of a tetrahedral open pore ferritin from the hyperthermophilic archaeon *Archaeoglobus fulgidus*. Structure.

[16] Sana B, Johnson E, Sheah K, Poh CL, Lim S (2010). Iron-based ferritin nanocore as a contrast agent. Biointerphases.

[17] Sana B, Johnson E, Lim S (2015). The unique self-assembly/disassembly property of *Archaeoglobus fulgidus* ferritin and its implications on molecular release from the protein cage. Biochim. Biophys. Acta.

[18] He DD, Marles-Wright J (2015). Ferritin family proteins and their use in bionanotechnology. N. Biotechnol..

[19] Milne JL, Wu X, Borgnia MJ, Lengyel JS, Brooks BR, Shi D, Perham RN, Subramaniam S (2006). Molecular structure of a 9-MDa icosahedral pyruvate dehydrogenase subcomplex containing the E2 and E3 enzymes using cryoelectron microscopy. J. Biol. Chem..

[20] Dalmau M, Lim S, Chen HC, Ruiz C, Wang SW (2008). Thermostability and molecular encapsulation within an engineered caged protein scaffold. Biotechnol. Bioeng..

[21] Ren D, Dalmau M, Randall A, Shindel MM, Baldi P, Wang SW (2012). Biomimetic design of protein nanomaterials for hydrophobic molecular transport. Adv. Funct. Mater..

[22] Domingo GJ, Orru S, Perham RN (2001). Multiple display of peptides and proteins on a macromolecular scaffold derived from a multienzyme complex. J. Mol. Biol..

[23] Kedersha NL, Rome LH (1986). Isolation and characterization of a novel ribonucleoprotein particle: large structures contain a single species of small RNA. J. Cell Biol..

[24] Poderycki MJ, Kickhoefer VA, Kaddis CS, Raval-Fernandes S, Johansson E, Zink JI, Loo JA, Rome LH (2006). The vault exterior shell is a dynamic structure that allows incorporation of vault-associated proteins into its interior. Biochemistry-US.

[25] Anderson DH, Kickhoefer VA, Sievers SA, Rome LH, Eisenberg D (2007). Draft crystal structure of the vault shell at 9-A resolution. PLoS Biol..

[26] Han M, Kickhoefer VA, Nemerow GR, Rome LH (2011). Targeted vault nanoparticles engineered with an endosomolytic peptide deliver biomolecules to the cytoplasm. ACS Nano.

[27] Kickhoefer VA, Han M, Raval-Fernandes S, Poderycki MJ, Moniz RJ, Vaccari D, Silvestry M, Stewart PL, Kelly KA, Rome LH (2009). Targeting vault nanoparticles to specific cell surface receptors. ACS Nano.

[28] Matsumoto NM, Prabhakaran P, Rome LH, Maynard HD (2013). Smart vaults: thermally-responsive protein nanocapsules. ACS Nano.

[29] Menon BB, Dou Z, Heinhorst S, Shively JM, Cannon GC (2008). *Halothiobacillus neapolitanus* carboxysomes sequester heterologous and chimeric RubisCO species. PLoS ONE.

[30] Yeates TO, Crowley CS, Tanaka S (2010). Bacterial microcompartment organelles: protein shell structure and evolution. Annu. Rev. Biophys..

[31] Valdes-Stauber N, Scherer S (1994). Isolation and characterization of Linocin M18, a bacteriocin produced by *Brevibacterium linens*. Appl. Environ. Microbiol..

[32] Yeates TO, Padilla JE (2002). Designing supramolecular protein assemblies. Curr. Opin. Struct. Biol..

[33] Padilla JE, Colovos C, Yeates TO (2001). Nanohedra: using symmetry to design self-assembling protein cages, layers, crystals, and filaments. Proc. Natl. Acad. Sci. U.S.A..

[34] Lai YT, King NP, Yeates TO (2012). Principles for designing ordered protein assemblies. Trends Cell Biol..

[35] Lai YT, Tsai KL, Sawaya MR, Asturias FJ, Yeates TO (2013). Structure and flexibility of nanoscale protein cages designed by symmetric self-assembly. J. Am. Chem. Soc..

[36] Bale JB, Gonen S, Liu Y, Sheffler W, Ellis D, Thomas C, Cascio D, Yeates TO, Gonen T, King NP, Baker D (2016). Accurate design of megadalton-scale two-component icosahedral protein complexes. Science.

[37] Gradišar H, Božič S, Doles T, Vengust D, Hafner-Bratkovič I, Mertelj A, Webb B, Šali A, Klavžar S, Jerala R (2013). Design of a single-chain polypeptide tetrahedron assembled from coiled-coil segments. Nat. Chem. Biol..

[38] Raja KS, Wang Q, Gonzalez MJ, Manchester M, Johnson JE, Finn MG (2003). Hybrid virus-polymer materials. 1. Synthesis and properties of PEG-decorated cowpea mosaic virus. Biomacromolecules.

[39] Lucon J, Qazi S, Uchida M, Bedwel GJ, LaFrance B, Prevelige PE, Douglas T (2012). Use of the interior cavity of the P22 capsid for site-specific initiation of atom-transfer radical polymerization with high-density cargo loading. Nat. Chem..

[40] Maham A, Tang Z, Wu H, Wang J, Lin Y (2009). Protein-based nanomedicine platforms for drug delivery. Small.

[41] Kohane DS, Langer R (2010). Biocompatibility and drug delivery systems. Chem. Sci..

[42] Naahidi S, Jafari M, Edalat F, Raymond K, Khademhosseini A, Chen P (2013). Biocompatibility of engineered nanoparticles for drug delivery. J. Control. Rel..

[43] Peng T, Lim S (2011). Trimer-based design of pH-responsive protein cage results in soluble disassembled structures. Biomacromolecules.

[44] Duncan R (2006). Polymer conjugates as anticancer nanomedicines. Nat. Rev. Cancer.

[45] Ashley CE, Carnes EC, Phillips GK, Durfee PN, Buley MD, Lino CA, Padilla DP, Phillips B, Carter MB, Willman CL, Brinker CJ, Caldeira, Jdo C, Chackerian B, Wharton W, Peabody DS (2011). Cell-specific delivery of diverse cargos by bacteriophage MS2 virus-like particles. ACS Nano.

[46] Volk AL, Rivera AA, Kanerva A, Bauerschmitz G, Dmitriev I, Nettelbeck DM, Curiel DT (2003). Enhanced adenovirus infection of melanoma cells by fiber-modification: incorporation of RGD peptide or Ad5/3 chimerism. Cancer Biol. Ther..

[47] Stevenson SC, Rollence M, White B, Weaver L, McClelland A (1995). Human adenovirus serotypes 3 and 5 bind to two different cellular receptors via the fiber head domain. J. Virol..

[48] Gillitzer, E., Willits, D., Young, M. & Douglas, T. Chemical modification of a viral cage for multivalent presentation. *Chem. Commun.* 2390–2391 (2002). 10.1039/b207853h 12430455

[49] Smith ML, Fitzmaurice WP, Turpen TH, Palmer KE (2009). Display of peptides on the surface of tobacco mosaic virus particles. Curr. Top. Microbiol. Immunol..

[50] Schlick TL, Ding Z, Kovacs EW, Francis MB (2005). Dual-surface modification of the tobacco mosaic virus. J. Am. Chem. Soc..

[51] Kovacs EW, Hooker JM, Romanini DW, Holder PG, Berry KE, Francis MB (2007). Dual-surface-modified bacteriophage MS2 as an ideal scaffold for a viral capsid-based drug delivery system. Bioconjugate Chem..

[52] Dedeo MT, Finley DT, Francis MB (2011). Viral capsids as self-assembling templates for new materials. Prog. Mol. Biol. Transl..

[53] Chen L, Zurita AJ, Ardelt PU, Giordano RJ, Arap W, Pasqualini R (2004). Design and validation of a bifunctional ligand display system for receptor targeting. Chem. Biol..

[54] Shin HI, Chae KH, Cho TJ (2013). Modification of Turnip yellow mosaic virus coat protein and its effect on virion assembly. BMB Rep..

[55] Zeng Q, Saha S, Lee LA, Barnhill H, Oxsher J, Dreher T, Wang Q (2011). Chemoselective modification of turnip yellow mosaic virus by Cu(I) catalyzed azide-alkyne 1,3-dipolar cycloaddition reaction and its application in cell binding. Bioconjugate Chem..

[56] Flenniken ML, Willits DA, Harmsen AL, Liepold LO, Harmsen AG, Young MJ, Douglas T (2006). Melanoma and lymphocyte cell-specific targeting incorporated into a heat shock protein cage architecture. Chem. Biol..

[57] Flenniken ML, Willits DA, Brumfield S, Young MJ, Douglas T (2003). The small heat shock protein cage from *Methanococcus jannaschii* is a versatile nanoscale platform for genetic and chemical modification. Nano Lett..

[58] Pan Y, Zhang Y, Jia T, Zhang K, Li J, Wang L (2012). Development of a microRNA delivery system based on bacteriophage MS2 virus-like particles. FEBS J..

[59] Zhao Q, Chen W, Chen Y, Zhang L, Zhang J, Zhang Z (2011). Self-assembled virus-like particles from rotavirus structural protein VP6 for targeted drug delivery. Bioconjugate Chem..

[60] Ma Y, Nolte RJ, Cornelissen JJ (2012). Virus-based nanocarriers for drug delivery. Adv. Drug Deliv. Rev..

[61] Schmidt U, Rudolph R, Bohm G (2001). Binding of external ligands onto an engineered virus capsid. Protein Eng..

[62] Seebeck FP, Woycechowsky KJ, Zhuang W, Rabe JP, Hilvert D (2006). A simple tagging system for protein encapsulation. J. Am. Chem. Soc..

[63] Fan CG, Cheng S, Liu Y, Escobar CM, Crowley CS, Jefferson RE, Yeates TO, Bobik TA (2010). Short N-terminal sequences package proteins into bacterial microcompartments. Proc. Natl. Acad. Sci. USA.

[64] Uchida M, Kang S, Reichhardt C, Harlen K, Douglas T (2010). The ferritin superfamily: Supramolecular templates for materials synthesis. Biochim. Biophys. Acta.

[65] Lin X, Xie J, Niu G, Zhang F, Gao H, Yang M, Quan Q, Aronova MA, Zhang G, Lee S, Leapman R, Chen X (2011). Chimeric ferritin nanocages for multiple function loading and multimodal imaging. Nano Lett..

[66] Goldsmith LE, Pupols M, Kickhoefer VA, Rome LH, Monbouquette HG (2009). Utilization of a protein ‘shuttle’ to load vault nanocapsules with gold probes and proteins. ACS Nano.

[67] Kickhoefer VA, Garcia Y, Mikyas Y, Johansson E, Zhou JC, Raval-Fernandes S, Minoofar P, Zink JI, Dunn B, Stewart PL, Rome LH (2005). Engineering of vault nanocapsules with enzymatic and fluorescent properties. Proc. Natl. Acad. Sci. USA.

[68] Ren D, Kratz F, Wang SW (2011). Protein nanocapsules containing doxorubicin as a pH-responsive delivery system. Small.

[69] Wu, W., Xing, L., Zhou, B. H. & Lin, Z. L. Active protein aggregates induced by terminally attached self-assembling peptide ELK16 in *Escherichia coli*. *Microb. Cell Fact* **10** (2011). 10.1186/1475-2859-10-9 PMC3045283 21320350

[70] Bode SA, Minten IJ, Nolte RJ, Cornelissen JJ (2011). Reactions inside nanoscale protein cages. Nanoscale.

[71] Abedin MJ, Liepold L, Suci P, Young M, Douglas T (2009). Synthesis of a cross-linked branched polymer network in the interior of a protein cage. J. Am. Chem. Soc..

[72] Peer D, Karp JM, Hong S, Farokhzad OC, Margalit R, Langer R (2007). Nanocarriers as an emerging platform for cancer therapy. Nat. Nanotechnol..

[73] Wu Y, Ng DY, Kuan SL, Weil T (2015). Protein–polymer therapeutics: a macromolecular perspective. Biomater. Sci..

[74] Domingo GJ, Caivano A, Sartorius R, Barba P, Bäckström M, Piatier-Tonneau D, Guardiola J, De Berardinis P, Perham RN (2003). Induction of specific T-helper and cytolytic responses to epitopes displayed on a virus-like protein scaffold derived from the pyruvate dehydrogenase multienzyme complex. Vaccine.

[75] Kang S, Suci PA, Broomell CC, Iwahori K, Kobayashi M, Yamashita I, Young M, Douglas T (2009). Janus-like protein cages. Spatially controlled dual-functional surface modifications of protein cages. Nano Lett..

[76] Suci PA, Kang S, Young M, Douglas T (2009). A streptavidin-protein cage Janus particle for polarized targeting and modular functionalization. J. Am. Chem. Soc..

[77] Tang J, Johnson JM, Dryden KA, Young MJ, Zlotnick A, Johnson JE (2006). The role of subunit hinges and molecular ‘switches’ in the control of viral capsid polymorphism. J. Struct. Biol..

[78] Zhang Y, Fu J, Chee SY, Ang EXW, Orner BP (2011). Rational disruption of the oligomerization of the mini-ferritin *E. coli* DPS through protein-protein interface mutation. Protein Sci..

[79] Dalmau M, Lim S, Wang SW (2009). Design of a pH-dependent molecular switch in a caged protein platform. Nano Lett..

[80] MacDonald JI, Munch HK, Moore T, Francis MB (2015). One-step site-specific modification of native proteins with 2-pyridinecarboxyaldehydes. Nat. Chem. Biol..

[81] Flenniken, M. L., Liepold, L. O., Crowley, B. E., Willits, D. A., Young, M. J. & Douglas, T. Selective attachment and release of a chemotherapeutic agent from the interior of a protein cage architecture. *Chem. Commun.* 447–449 (2005). 10.1039/b413435d 15654365

[82] Yang, Z., Wang, X., Diao, H., Zhang, J., Li, H., Sun, H. & Guo, Z. Encapsulation of platinum anticancer drugs by apoferritin. *Chem. Commun.* 3453–3455 (2007). 10.1039/b705326f 17700879

[83] Fang CY, Lin PY, Ou WC, Chen PL, Shen CH, Chang D, Wang M (2012). Analysis of the size of DNA packaged by the human JC virus-like particle. J. Virol. Methods.

[84] Choi KM, Kim K, Kwon IC, Kim IS, Ahn HJ (2013). Systemic delivery of siRNA by chimeric capsid protein: tumor targeting and RNAi activity *in vivo*. Mol. Pharmaceut..

[85] Kaczmarczyk SJ, Sitaraman K, Young HA, Hughes SH, Chatterjee DK (2011). Protein delivery using engineered virus-like particles. Proc. Natl. Acad. Sci. USA.

[86] Lee H, Song JJ, Kim E, Yun C-O, Choi J, Lee B, Kim J, Chang JW, Kim J-H (2001). Efficient gene transfer of VSV-G pseudotyped retroviral vector to human brain tumor. Gene Therapy.

[87] Lakshmanan A (2016). Molecular Engineering of Acoustic Protein Nanostructures. ACS Nano.

[88] Hooker JM, O'Neil JP, Romanini DW, Taylor SE, Francis MB (2008). Genome-free viral capsids as carriers for positron emission tomography radiolabels. Mol. Imag. Biol..

[89] Harrison PM, Arosio P (1996). The ferritins: molecular properties, iron storage function and cellular regulation. Biochim. Biophys. Acta.

[90] Champion CI, Kickhoefer VA, Liu G, Moniz RJ, Freed AS, Bergmann LL, Vaccari D, Raval-Fernandes S, Chan AM, Rome LH, Kelly KA (2009). A vault nanoparticle vaccine induces protective mucosal immunity. PLoS ONE.

[91] Kanekiyo M, Wei CJ, Yassine HM, McTamney PM, Boyington JC, Whittle JR, Rao SS, Kong WP, Wang L, Nabel GJ (2013). Self-assembling influenza nanoparticle vaccines elicit broadly neutralizing H1N1 antibodies. Nature.

[92] Plummer EM, Manchester M (2011). Viral nanoparticles and virus-like particles: platforms for contemporary vaccine design. Wiley Interdiscip. Rev..

[93] Farris CM, Morrison RP (2011). Vaccination against Chlamydia genital infection utilizing the murine *C. muridarum* model. Infect. Immun..

[94] Akahata W, Yang ZY, Andersen H, Sun S, Holdaway HA, Kong WP, Lewis MG, Higgs S, Rossmann MG, Rao S, Nabel GJ (2010). A virus-like particle vaccine for epidemic Chikungunya virus protects nonhuman primates against infection. Nat. Med..

[95] Quan FS, Kim Y, Lee S, Yi H, Kang SM, Bozja J, Moore ML, Compans RW (2011). Virus like particle vaccine induces protection against respiratory syncytial virus infection in mice. J. Infect. Dis..

[96] Murawski MR, McGinnes LW, Finberg. RW, Kurt-Jones EA, Massare MJ, Smith G, Heaton PM, Fraire AE, Morrison TG (2010). Newcastle disease virus-like particles containing respiratory syncytial virus g protein induced protection in BALB/c mice, with no evidence of immunopathology. J. Virol..

[97] Zhu Y, Jiang J, Said-Sadier N, Boxx G, Champion C, Tetlow A, Kickhoefer VA, Rome LH, Ojcius DM, Kelly KA (2015). Activation of the NLRP3 inflammasome by vault nanoparticles expressing a chlamydial epitope. Vaccine.

